# Clinical effect of pricking blood at neiyingxiang (ExHN 9) on non-allergic rhinopathy: study protocol for a randomized controlled trial

**DOI:** 10.1186/1745-6215-13-37

**Published:** 2012-04-16

**Authors:** Kyu Seok Kim, Sang Gyu Go, Yoon-Bum Kim

**Affiliations:** 1 Department of Ophthalmology & Otolaryngology & Dermatology, College of Oriental Medicine, Kyung Hee University, 1 Hoegi-dong, Dongdaemun-gu, Seoul 130-701, Republic of Korea; 2 Copyeonhan Korean medical clinic, #201, Samsung-chereville, 319-12, Sinjung-dong, Yangcheon-ru, Seoul 158-764, Republic of Korea

## Abstract

**Background:**

Non-allergic rhinopathy (NAR), formerly known as vasomotor rhinitis, is a non-allergic and non-infectious chronic disease that is accompanied by nasal hyperemia, rhinorrhea, and no increase in the number of eosinophils. Although the medications for NAR, including intranasal corticosteroids and intranasal antihistamine, have been used in clinical practice, given the relative paucity of effective therapy with available medications, alternative non-pharmacologic treatments could play an important role in treating NAR. Acupuncture treatment is representative potential alternative therapy for the treatment of various diseases, including rhinitis. Therefore, the objective of this study was to evaluate the efficacy of pricking blood at Neiyingxiang (ExHN 9) relative to acupuncture treatment at Waiyingxiang (LI 20) in patients with NAR.

**Methods/Design:**

A randomized, parallel-group, controlled, assessor single-blinded, trial will be conducted. Fifty participants with NAR will be randomized into one of two groups: either the control group with acpuncture treatment at LI 20 or the experimental group with pricking blood at ExHN 9. After randomization, a total of three sessions of treatment will be performed once a week in both groups. The total nasal symptom score (TNSS) and the Mini-Rhinoconjunctivitis Quality-of-Life Questionnaire (MiniRQLQ) at baseline and the end of the trial will be used to evaluate the efficacy of each treatment.

**Discussion:**

This study will be the first randomized trial to evaluate the efficacy of pricking blood for the treatment of NAR. The results of this study will help establish an alternative approach for treating patients with NAR that do not respond to Western medication.

**Trial registration:**

The trial was registered with the Clinical Research Information Service (CRiS), Republic of Korea: KCT0000195.

## Background

Non-allergic rhinopathy (NAR), formerly known as vasomotor rhinitis, is a chronic non-infectious and non-allergic condition that displays the following symptoms: predominantly nasal congestion and rhinorrhea, which may be perennial, persistent, intermittent, or seasonal [1,2]. Unlike allergic rhinitis (AR), which is elicited by specific antigens, NAR is not relevant to allergen exposure and may be triggered by cold air, changes in climate or sexual hormone levels, strong smells, environmental tobacco smoke, pollutants, chemicals, exercise, or alcohol ingestion [1]. The exact prevalence of NAR has not been as well established as allergic rhinitis and one study suggested a relative prevalence rate of 76% for AR and 24% for non-allergic rhinitis, which corresponds to an approximate ratio of 3:1 [1,3].

The medications used for treating NAR have been studied less widely than those for AR [1]. The management of NAR consists of avoiding trigger factors in combination with medication, including intranasal corticosteroids, intranasal antihistamines, anti-cholinergics, decongestants, and nasal saline [1-5]. Even though patients with NAR are administered these medications, some patients do not adequately respond to them. For these patients, other agents can be considered such as capsaicin, botulinum toxin A, antileukotrienes, silver nitrate, or acupuncture [1,3]. However, given the relative paucity of good therapeutic agents with available medications, alternative non-pharmacologic therapies could play an important role in treating NAR.

Acupuncture has been used widely in East Asia and Europe for the treatment of various kinds of diseases, including rhinitis. In a systematic review that evaluated the clinical effectiveness of acupuncture for treating or preventing allergic rhinitis in 2009, acupuncture was found to be ineffective in the treatment of seasonal AR; however, acupuncture was shown to be effective for the treatment of perennial AR [6]. Recently, acupuncture treatment was shown to be effective in improving the symptoms of patients with persistent allergic rhinitis complicated by rhinosinusitis and asthma [7]. However, the majority of studies on acupuncture treatment have only examined AR and there were seldom randomized controlled trials. There has only been one randomized controlled pilot trial that showed a significant change in the nasal sickness score of patients with vasomotor rhinitis [8].

In Oriental medicine, Waiyingxiang (LI 20) is generally used in clinical practice to treat local diseases of the nose including rhinitis, sinusitis, and olfactory disorders [7-12]. Neiyingxiang (ExHN 9) is located at the junction between the mucosa of the alar cartilage of the nose and the nasal concha in the nostril and is commonly used for olfactory disorders, chronic rhinitis, sequela of Bell's palsy, and headache [13-15].

Therefore, we will conduct a randomized controlled pilot trial to assess the efficacy of pricking blood at ExHN 9 relative to acupuncture treatment at the LI 20 in patients with NAR.

## Method/Design

### Objective

The current randomized, assessor single-blinded, parallel-group, single-center study was designed to evaluate the clinical efficacy of pricking blood at ExHN 9 for the treatment of NAR relative to the control group, which involved administration of acupuncture treatment at LI 20.

### Design

**Figure 1:**
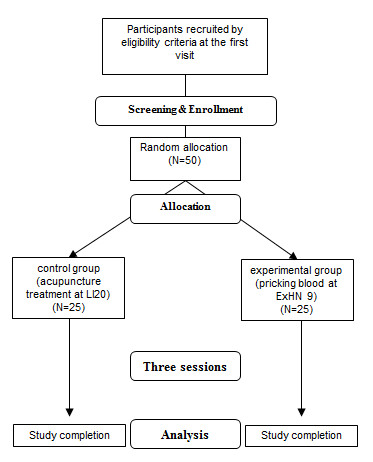
**Flow chart of the study**.

This study will be a randomized, real acupuncture-controlled, assessor single-blinded and single-center pilot trial. The study will be conducted in the following order: enrollment after screening via inclusion and exclusion criteria, randomization, a treatment period of 3 weeks, and assessment (Figure 1).

### Participants and eligibility

#### Inclusion criteria

Eligible for inclusion in this study will be patients ≥ 8 years of age with a diagnosis of NAR and at least a 3-month history of continuous nasal symptoms related to defined triggers (for example, changes in temperature and/or humidity, strong odors, and airborne irritants). Patients must have had a history of negative results in allergic tests (MAST test, serum IgE) and an eosinophil count within a normal range.

#### Exclusion criteria

Patients that had a history of nasal surgery, nasal polyp, psychiatric disorders, drug addiction, and autoimmune disease will be excluded, as well as patients that could not be subjected to acupuncture treatment, such as individuals that were pregnant or had a blood clotting disorder. Participants will be ineligible if they have undergone acupuncture treatment or Western medical treatment for rhinitis within 1 month of taking cold medicine, within 2 weeks prior to registration, or if cold medicine was taken during the trial. Patients who were treated with gastritis, gastric ulcer (using H2 blocker antiacid), who had plans to participate in another clinical trial during this clinical trial, who participated in another clinical trial within 6 months, or who are not willing to not comply with the protocol of this study protocol will also be excluded. Within 3 weeks after enrollment in this trial, no concomitant therapy will be permitted.

#### Recruitment

Participants will be recruited via an internet advertisement posted on the website of the Kyung Hee Medical Center and via ad-posters.

#### Randomization and blinding

After enrollment, participants will be randomly assigned to control and experimental groups. The allocation ratio will be 1:1. Randomization will be performed at a site remote from trial location. Random numbers will be generated using a computerized random-number generator through the block-randomization method (Excel, Microsoft Office 2007) for sequence generation. Two separate databases will be generated: a 'patient' database, which lists basic information, such as the patient name, contact details, and so on, and a 'randomization' database, which contained data on patients that have been registered in the trial along with their allocations. The 'patients' database will be accessible to any researcher whereas the 'randomization' database will be password-protected so that it will be accessible only by the principal investigator and a nominated statistician [16]. The assessor, who will not be the acupuncture practitioner, and who will be blinded to the allocation results until the end of study, will assess the outcome of treatment.

#### Intervention

Three sessions will be used for both pricking the blood at ExHN 9 and acupuncture treatment at LI 20. The sessions will be administered over a period of 3 weeks (one session per week). Certified acupuncture practitioners who have a minimum of 3 years of clinical experience obtained after a 6-year oriental medical college course will perform the acupuncture treatment. The practitioners will take a one-day training course for this trial. This course will include the study protocol, methods for acupuncture treatment, and basic information on clinical research.

#### Pricking blood at ExHN 9

Three sessions of pricking blood at ExHN will be performed once per week for 3 weeks in patients assigned to the experimental group. Patients that will receive acupuncture treatment will be pricked on the intranasal membrane at bilateral ExHN 9, which is located at the junction between the mucosa of the alar cartilage of the nose and the nasal concha in the nostril. This point will be treated with 0.30 × 120 mm disposable acupuncture needles (Dongbang Co., Korea) according to 'WHO Standard Acupuncture Point Locations' in the Western Pacific Region [17].

#### Acupuncture treatment at LI 20

Three sessions of acupuncture treatment at LI 20 will be performed once per week for 3 weeks in patients assigned to the control group. Patients will receive acupuncture treatment at bilateral LI 20. For bilateral LI 20, needles will be inserted obliquely toward their ipsilateral nostrils to a depth of 0.3 cm. This point will be treated with 0.30 × 40 mm disposable acupuncture needles (Dongbang Co., Korea) according to 'WHO Standard Acupuncture Point Locations' in the Western Pacific Region [17]. Patients will remain in the supine position for 15 min during acupuncture treatment.

### Outcome measures

#### Primary outcome

Primary outcomes will be presented by mean change in total nasal symptom score (TNSS) from baseline to the end of the trial. TNSS is a scoring system that consisted of rhinorrhea, sneezing, nasal itching, and nasal obstruction, which are scored on a severity scale from 0 to 3 (0 = none, 1 = mild, 2 = moderate, and 3 = severe). The TNSS is calculated by summation of all four symptom scores [5,7]. For each visit at baseline and the end of trial, the patient will record his nasal symptoms using the TNSS scoring system.

#### Secondary outcomes

Secondary outcomes will be evaluated using the Mini-Rhinoconjunctivitis Quality-of-Life Questionnaire (MiniRQLQ).

#### MiniRQLQ

The MiniRQLQ consists of five domains with a total of 14 questions: two questions for practical problems, and three questions each for activity limitations, nose, eye, and other symptoms [18]. The MiniRQLQ is a self-administered scoring system. The patients will mark one of the seven Likert-like scales (0 = no impairment to 6 = maximum impairment) for each of the 14 questions.

### Statistical methods

#### Statistical analysis plan

Statistical analysis will be conducted on an intention-to-treat basis with a 95% confidence interval using SPSS version 12.0 for Windows. Missing values for patients that drop out of the study will be analyzed using the last observation carried forward (LOCF) method. Data will be displayed as the mean ± standard deviation (SD) for continuous data or *n *(%) for categorical data. To control for baseline differences between groups, variables that are significantly different at baseline will serve as covariates in the analyses.

#### Demographic and clinical characteristics

Demographic and clinical characteristics of participants in the control and experimental group will be compared upon admission using an independent sample *T*-test (continuous data) and chi-square analysis (categorical data).

#### Efficacy analysis

The statistical calculation for the paired sample *T*-test or independent sample *T*-test will be done for the mean change of TNSS and miniRQLQ score in comparison between and within groups from baseline to the end of trial. Non-parametric methods will be used when assumptions of normality are violated.

#### Adverse events and monitoring safety

All unexpected adverse events related to pricking blood at ExHN 9 and acupuncture treatment at LI 20 will be reported to the investigator or acupuncture practitioner by participants and written on the individual case report form by the investigator. Safety will be assessed using clinical laboratory tests, vital sign measurements, and adverse events. Clinical laboratory tests, including AST/ALT, BUN/creatinine, red blood cell (RBC) count, white blood cell (WBC) count, hemoglobin, hematocrit, mean cell volume (MCV), mean cell hemoglobin (MCH), mean cell hemoglobin concentration (MCHC), number of platelets, and number of differentiated cells will be determined at weeks 0 (baseline) and 3 (end of the trial). Vital signs of each participant will be checked by monitoring adverse events (pain on the acne lesion or other sites, nausea/vomiting, fatigue, allergic reaction, and any adverse events related to acupuncture) after each visit.

#### Sample size

This study is a pilot study to evaluate the clinical effects of pricking blood at ExHN 9 on NAR. Because this trial will be designed for a short duration, lasting 3 weeks, with the intention of decreasing the drop-out rate, the desired sample size for this pilot study is 50 patients, with 25 for each group, assuming a drop-out rate of 20%.

## Discussion

A placebo-control group is a decisive tool to evaluate whether the clinical effectiveness of an intervention is truly due to the characteristic elements of an intervention [19]. Even though several recent large randomized trials found clinically relevant effects of acupuncture over no treatment or routine care, blinded trials comparing acupuncture to sham interventions often reported only minor or no differences [20]. A recent Cochrane review on placebo interventions for all clinical conditions showed that placebo interventions have no important clinical effects in general, but in certain settings, placebo interventions can influence patient-reported outcomes and physical placebos including sham acupuncture were associated with larger effects over no-treatment control groups than pharmacological placebos [21]. Thus, in trials to evaluate the clinical effect of acupuncture treatment, a control group must be included because a sham or placebo control can affect outcomes in certain designed settings. In particular, in Korea, several patients have used acupuncture treatment and are familiar with acupuncture treatment. Because it is difficult to apply a placebo-control, including sham acupuncture, to Korean patients, only minor or no differences are often reported in blinded trials that compare acupuncture to sham interventions [20]. In this study, we will use real acupuncture treatment as the control group. Acupuncture treatment at LI 20 is the most common therapy used to treat patients with nasal disease in the clinical practice of Oriental medicine [9-12]. In this study, the effect of pricking blood at ExHN 9 which is modified acupuncture treatment method, to reduce the nasal congestion and hypersensitivity will be evaluated [13-15]. Therefore, acupuncture treatment at LI 20 will be used as the control group to access the effectiveness of pricking blood at ExHN 9.

The primary goal of treating NAR patients is to give symptomatic relief. So, the primary outcome that will be evaluated in this study is the mean change in TNSS from baseline to the end of the trial. Since NAR is a condition that markedly affects the quality of life, we will also examine the mean change in MiniRQLQ scores from baseline to the end of trial as a secondary outcome.

This study will be the first randomized trial to evaluate the efficacy of pricking blood at ExHN 9 for the treatment of NAR. The main focus of this study is to investigate the effectiveness of pricking blood at ExHN 9 in decreasing the TNSS, as a primary outcome, relative to acupuncture treatment at LI 20, which is commonly used in clinical practice and has been shown to be effective in treating patients with chronic rhinitis including NAR. This trial will provide information needed to answer relevant clinical questions regarding the efficacy of pricking blood at ExHN 9 for the treatment of NAR. Although the size of this trial is small and cannot provide conclusive results on the effectiveness of pricking blood at ExHN 9, if positive results are obtained, a large clinical trial will be conducted in the future.

## Trial status

Not yet recruiting.

## Ethics

The study protocol and the written informed consent were approved by the institutional review board (IRB) of the Oriental Medical Hospital at Kyung Hee Medical Center (KOMC MIRB 2010-04). Each participant will be notified regarding the study protocol. Written informed consent will be obtained from each patient.

## Abbreviations

ALT: Alanine transaminase; AST: Aspartate transaminase; BUN: Blood urea nitrogen; ExHN 9: Neiyingxiang; HRV: Heart rate variation; IRB: Institutional review board; LI 20: Waiyingxiang; LOCF: Last observation carried forward; MAST: Multiple antigen simultaneous test; MCH: Mean cell hemoglobin; MCHC: Mean cell hemoglobin concentration; MCV: Mean cell volume; MiniRQLQ: Mini-rhinoconjunctivitis quality-of-life questionnaire; NAR: Non-allergic rhinopathy; RBC: Red blood cell; RCT: Randomized controlled trial; SD: Standard; WBC: White blood cell.

## Competing interests

The authors declare that they have no competing interests.

## Authors' contributions

KSK participated in the study design, including statistical design, and drafted the manuscript. SGG participated in the study design and critical revision of the manuscripts. YBK was the general supervisor for this research and participated in both the study design and critical revision of the manuscript. All authors read and approved the final manuscript.
